# Inhibition of efflux pumps by FDA-approved drugs oxiconazole and sertaconazole restores antibiotic susceptibility in multidrug-resistant *S. aureus*

**DOI:** 10.1128/aac.00320-25

**Published:** 2025-08-04

**Authors:** Suvendu Ojha, Puja Chatterjee, Tushar Kant Beuria

**Affiliations:** 1 Department of Infectious Disease Biology, Institute of Life Sciences 29722, Bhubaneswar, Odisha, India; 2 Regional Centre for Biotechnology 214253, Faridabad, Haryana, India; The Peter Doherty Institute for Infection and Immunity, Melbourne, Victoria, Australia

**Keywords:** efflux pump, EPI, *S. aureus*, multidrug resistance, sertaconazole, oxiconazole, MRSA, PMF inhibitor

## Abstract

Antibiotic resistance in *Staphylococcus aureus* causes major concern worldwide. In *S. aureus*, efflux pumps are mostly responsible for the development of multidrug resistance. Active removal of antibiotics from cells by *S. aureus* efflux pumps, including NorA, NorB, AbcA, and MepA, helps to lower their intracellular concentration and effectiveness. The present study examined two FDA-approved antifungal medications, oxiconazole and sertaconazole, as possible efflux pump inhibitors (EPIs) against multidrug-resistant *S. aureus*. Our results showed that both drugs reduced the efflux pump activity of drug-susceptible (ATCC25923) and multidrug-resistant (Mu50) *S. aureus* strains. While sertaconazole inhibited the efflux pumps without changing the efflux rate, oxiconazole lowered both efflux pump activity and efflux rate. Neither of these drugs impacted bacterial membrane integrity nor bacterial growth. Both drugs enhanced the efficacy of norfloxacin, cefotaxime, and moxifloxacin by lowering the MIC values and showed minimal cytotoxicity toward mammalian cells. In combination with the antibiotics, both sertaconazole and oxiconazole significantly lowered the bacterial load in a murine skin infection model. Our results suggested that the drugs altered the proton motive force (PMF), which resulted in diminished membrane potential (Δ*Ψ*) and an increased electrochemical gradient (ΔpH), thereby inhibiting ATP production and efflux pump activity. The safety profile and potential to enhance antibiotic efficacy suggest that sertaconazole and oxiconazole may be used as EPIs for combating multidrug-resistant *S. aureus* infections. Further studies are required to assess their pharmacokinetics, toxicity, and activity against a wide range of *S. aureus* isolates.

## INTRODUCTION

*Staphylococcus aureus*, a gram-positive bacterium, poses a significant public health concern due to its widespread presence and ability to cause various infections (1, 2). Estimates suggest that approximately 50% of adults are either permanent or intermittent carriers of *S. aureus*, highlighting the organism’s widespread presence in the human population (3). *S. aureus* can cause various infections like impetigo, folliculitis, infected wounds, and bacteremia, potentially resulting in critical conditions like sepsis (1, 2). One of the most concerning developments in *S. aureus* epidemiology is the emergence and spread of methicillin-resistant *S. aureus* (MRSA) strains due to overuse of antibiotics. MRSA strains often show multidrug resistance, complicating effective treatment with conventional antibiotics and emphasizing the necessity for an effective medical approach to address this public health issue (4–7).

Bacteria have developed various methods to resist the effects of antibiotics, allowing them to survive in the presence of antibiotics. One such method is to enhance the expression or activity of efflux pumps to remove antibiotics from the cell to decrease the intracellular concentration of antibiotics, resulting in a multidrug-resistant phenotype (8–11). Efflux pumps in bacteria are classified into five primary categories: the ATP-Binding Cassette (ABC) superfamily, the Major Facilitator Superfamily (MFS), the Resistance-Nodulation-Division (RND) family, the Small Multidrug Resistance (SMR) family, and the Multidrug and Toxic Compound Extrusion (MATE) family. The ABC superfamily uses energy from ATP hydrolysis to facilitate substrate efflux, while the MFS, SMR, and RND superfamilies utilize the proton motive force (PMF), and the MATE superfamily utilizes sodium gradients as its energy source (10, 12, 13). The electrochemical gradient (ΔpH) and the electric or membrane potential (Δ*Ψ*) constitute the proton motive force (PMF) in bacteria. The unequal distribution of H^+^ ions across the bacterial membrane produces the ΔpH, whereas the unequal distribution of other ions, including K^+^, Cl⁻, and Na^+^, across the bacterial membrane contributes to the membrane potential (Δ*Ψ*). The bacterial ATP synthase utilizes the energy produced by the unequal distribution of H^+^ ions across the bacterial membrane to produce ATP (14–20).

The primary efflux pumps identified in *S. aureus* include NorA, NorB, QacA, and QacB, which are classified under the MFS type. Additionally, AbcA, Sav1866, and MsrA are categorized as ABC types, while MepA is associated with the MATE type, and Smr is part of the SMR family (10, 21, 22). The resistance of *S. aureus* to fluoroquinolones (norfloxacin and moxifloxacin) is typically driven by the overexpression of NorA and NorB efflux pumps, while the resistance to cephalosporins (cefotaxime) is associated with the overexpression of AbcA efflux pumps (10, 23). The efflux-mediated resistance can be prevented by efflux pump inhibitors (EPIs) through (a) modifying gene regulation to decrease the expression of efflux genes, (b) changing the structure of antibiotics so they are not recognized as substrates, (c) preventing the assembly of the efflux pumps, (d) inhibiting the binding of substrates to the efflux pumps, and (e) inhibiting the energy source necessary for the efflux pump. Since EPIs inhibit the efflux pumps, they can enhance the efficacy of antibiotics by increasing cytoplasmic retention and potentially be used as adjuvants (24–28).

EPIs against *S. aureus* have been identified from both natural and synthetic origins. For example, plant-derived EPIs, such as reserpine, curcumin, silibinin, coumarins, etc. (21, 26, 29, 30), and synthetic molecules such as 4-acetyl-3-(4-fluorophenyl)-1-(p-tolyl)-5-methylpyrrole, N-trans-3,4-O-dimethylcaffeoyl tryptamine, etc., inhibit the NorA efflux pump (21, 26, 29, 30). Furthermore, the plant-derived EPI Clerodane diterpene 16α-hydroxycleroda-3,13 (14)-Z-dien-15,16-olide inhibits the NorB, NorC, MepA, and MdeA efflux pumps. Linoleic and oleic acids inhibit the MsrA efflux pump; α-terpinene blocks the TetK efflux pump. Likewise, synthetic EPIs such as 10-(4-(-3-phenylureido)-benzylamino)-9-fluoro-3,7-dihydro-3-methyl-7-oxo-2H-[1,4]oxazino[2,3,4-ij]-Quinoline-6-carboxylic acid (Q6CA) inhibits both the NorA and MepA efflux pumps, whereas cholecalciferol and alpha-tocopherol inhibit the TetK and MsrA efflux pumps (21, 26, 29, 30). However, none of these EPIs have reached successful commercialization, mostly because of their high cytotoxicity and the complexities associated with their synthesis (21, 26, 29, 30). Furthermore, in recent times, to address the challenges of natural and synthetic EPIs, FDA-approved drugs have been repurposed as EPIs. Drugs such as nilotinib, diclofenac sodium, domperidone, and glyceryl trinitrate have been identified as EPIs against *S. aureus*, specifically targeting the NorA efflux pump. While these EPIs show promise, their action is limited primarily to NorA, and their potency is suboptimal, exhibiting only moderate inhibition of efflux pumps (13, 31–33). The current study reveals two FDA-approved antifungal drugs, sertaconazole and oxiconazole, as effective EPIs against *S. aureus*. Both EPIs exhibited minimal cytotoxicity toward mammalian cells and synergistically improved the efficacy of antibiotics significantly against multidrug-resistant *S. aureus* infections in both *in vitro* and murine skin infection models. The findings further indicated that sertaconazole and oxiconazole inhibited efflux pumps by disrupting the energy necessary for their activity.

## MATERIALS AND METHODS

### Materials

Ethidium bromide (EtBr, Product Number E1510), chlorpromazine (CPZ, Product Number C8138), thioridazine (TZ, Product Number 1662504), glucose (glu, Product Number G8644), and cefotaxime sodium (Cefo, Product Number 1097909) from Sigma-Aldrich. Norfloxacin (Nor, Item No. 25975), moxifloxacin (Mox, Item No. 14830), sertaconazole nitrate (Sert, Item No. 22232), and oxiconazole nitrate (Oxi, Item No. 18724) were purchased from Cayman Chemicals. Tryptone soya agar (TSA, Catalogue No. M1968), tryptone soya broth (TSB, Catalogue No. M011), antibiotic E-strips, and mannitol salt agar (MSA, Catalogue No. MH118) were obtained from Himedia. Additionally, the Bacterial Membrane Potential Assay Kit (Catalogue Number: B349500) and the Live-Dead Assay Kit (Catalogue Number: L7012) were received from Invitrogen.

### Bacterial strains, plasmids, and culture conditions

The bacterial strains studied are methicillin-sensitive *Staphylococcus aureus *subsp*. aureus *Rosenbach (MSSA, ATCC25923) and multidrug-resistant *Staphylococcus aureus *subsp*. aureus *strain Mu50 (NRS1, ATCC700699). The cells were grown in a TSB medium at 37°C and 200 rpm. The bacterial growth phase was determined by changes in optical density at 600 nm (OD_600_) using a Shimadzu spectrophotometer.

### Ethidium bromide accumulation assay

As previously described, fluorometry was used to measure the accumulation of EtBr in both the ATCC25923 and Mu50-ATCC700699 strains (34, 35). The bacterial strains were cultured until OD_600_ ~ 0.6. Subsequently, the cells were centrifuged and resuspended to an OD_600_ ~ 0.3 using phosphate-buffered saline (PBS), pH 7.4. The OD-adjusted cells were seeded in a 96-well black plate. EtBr at 1 µg/mL was added to all experimental groups, while sertaconazole and oxiconazole were used at a final concentration of 10 µM (~4.9 µg/mL). TZ, a recognized efflux pump inhibitor, was used at half MIC concentration (16 µg/mL). At the same time, glucose (0.4%) was used as a negative control due to its role in enhancing efflux activity. All treatments were performed simultaneously during the addition of EtBr. Finally, fluorescence intensity (FI) changes were measured at 37°C and 200 rpm for 1 h at 5 min intervals in a VictorNivo multimode plate reader (PerkinElmer, RRID: SCR_025763). The FI was measured at an excitation wavelength of 530 nm and an emission wavelength of 585 nm. The OD_600_ adjusted fluorescence intensity at 60 min was chosen for the calculations of EtBr accumulation.

### Ethidium bromide efflux assay

The effects of sertaconazole and oxiconazole on EtBr efflux were examined in both ATCC25923 and Mu50 using a fluorimeter, as previously described (34–36). In an approach similar to the EtBr accumulation assay, the OD-adjusted cells (OD_600_ ~ 0.3) were subjected to incubation with EtBr (1 µg/mL) together with sertaconazole (10 µM), oxiconazole (10 µM), CPZ (16 µg/mL), and TZ (16 µg/mL) across various experimental groups for 1 h at 37°C and 200 rpm. Following a 1 h incubation period to activate the efflux, the cells were treated with 0.4% glucose. The glucose-treated cells were then assessed for changes in FI at 530 nm excitation and 585 nm emission wavelengths at 5 min intervals for 1 h using a VictorNivo multimode plate reader. The OD-adjusted FI of cells at 60 min was selected to calculate EtBr efflux.

### Bacterial susceptibility testing

Following the guidelines established by the Clinical and Laboratory Standards Institute (CLSI) (37) and the European Committee on Antimicrobial Susceptibility Testing (EUCAST) (38), the minimum inhibitory concentration (MIC) and minimum bactericidal concentration (MBC) of the antibiotics (norfloxacin, moxifloxacin, and cefotaxime), sertaconazole, and oxiconazole were assessed using the broth microdilution method using a previously standardized protocol (39). Briefly, bacterial cells were cultured to an OD_600_ ~ 1; working concentrations of the antibiotics, sertaconazole and oxiconazole, were prepared at 256 µg/mL in TSB media. Using the serial dilution method, a concentration range of 128–0.125 µg/mL was prepared from the working concentration in a 96-well microtiter plate (Eppendorf, Catalogue Number: CLS9017). Finally, bacterial cultures were added at a final concentration of 0.5 MacFarland standard, and the plate was incubated for 18 h at 37°C and 200 rpm. After 18 h of incubation, the plates were assessed for a change in turbidity, and MIC was identified as a concentration with no turbidity. Subsequently, from the 18 h incubated plate, 3 µL was inoculated onto TSA plates and incubated for 18 h at 37°C to assess MBC concentration. After 18 h of incubation, plates were analyzed, and MBC was determined as the concentration with no bacterial colonies (39).

### Synergy assay

The potential synergistic interaction between sertaconazole and oxiconazole to increase the *in vitro* effectiveness of existing antibiotics (norfloxacin, moxifloxacin, and cefotaxime) against ATCC25923 and Mu50 was studied using the previously mentioned checkerboard synergy assay with modifications (40, 41). Bacterial cells were cultured until OD_600_ ~ 1. Working concentrations (10× of the final required concentration) of antibiotics, sertaconazole, and oxiconazole were prepared in TSB and added onto the plate in various combinations. Bacterial cultures were added to each well at a final concentration of 0.5 MacFarland standard and incubated at 37°C and 200 rpm for 18 h. The plates were analyzed after 18 h to observe the change in turbidity, and the lowest combined concentration that exhibited no turbidity was determined as the MIC in combination. The fractional inhibitory concentration (FIC) was evaluated using the MIC values obtained from susceptibility testing (MIC alone) and synergy assays (MIC in combination). The FIC was calculated as mentioned below. Where A represents antibiotics, and B represents sertaconazole or oxiconazole. The combination was assessed as synergistic when FIC ≤ 0.5, additive when FIC > 0.5 and <1, indifferent when FIC > 1 and <4, and antagonistic when FIC ≥ 4.


FIC=FICA+FICB

FICA=(MIC of A in combination) ÷(MIC of A alone)

FICB=(MIC of B in combination) ÷(MIC of B alone)

### Bacterial growth curve

Following the previously described bacterial growth curve assay (42), we assessed the effect of sertaconazole and oxiconazole on bacterial growth. In the process of growth curve analysis, bacterial cells were cultured overnight. Subsequently, a fresh inoculum of a 0.5 McFarland standard culture was prepared in TSB by diluting the overnight culture within a 48-well microtiter plate. Sertaconazole and oxiconazole were given at various time intervals at a final concentration of 4 µg/mL. The plate was observed for changes in OD_600_ with a VictorNivo multimode plate reader every hour for 6 h, maintained at 37°C and 200 rpm. The OD_600_ readings were analyzed to observe the effect of sertaconazole and oxiconazole on bacterial growth.

### Live-dead assay and microscopy

The cell viability of bacteria was assessed using flow cytometry (BD Accuri C6 Plus, RRID: SCR_014422) and fluorescence microscopy with the LIVE/DEAD BacLight Bacterial Viability and Counting Kit. Fluorescence microscopy was conducted according to a previously standardized method (42, 43). Flow cytometry was performed according to the manufacturer’s protocol. Both studies used a concentration of 4 µg/mL. Flow cytometry observations were recorded in logarithmic mode during acquisition and analyzed utilizing FlowJo software (RRID: SCR_008520). In flow cytometry, an unstained control was utilized to gate the target cells. In contrast, an untreated culture (ATCC25923 or Mu50) was used as the live cell control in both studies, and a culture treated with 70% ethanol (Ethanol) was used as the dead cell control. Initially, unstained cells were gated in flow cytometry based on forward scatter (FSC) and side scatter (SSC) characteristics to distinguish the target cells and eliminate any cell debris. The target cells were subsequently gated according to the fluorescence of Syto9 and PI to identify live and dead cell populations, respectively.

### Cytotoxicity assay

The MTT cell viability assay was performed according to the manufacturer’s guidelines to study the effect of sertaconazole and oxiconazole on the viability of mammalian cells, either alone or in combination with antibiotics. The study was performed on human lung cancer adherent cells (A549, RRID: CVCL_UJ49) and kidney epithelial cells (HEK293T, RRID: CVCL_0063). For the individual effect of sertaconazole or oxiconazole, the change in cell viability was assessed by comparing the absorbance at 570 nm with non-treated cells (control). For the combinatorial effect of sertaconazole or oxiconazole with antibiotics, the cell viability was assessed by comparing the absorbance at 570 nm with individually treated cells (control). The 50% cytotoxic concentration (CC_50_) of sertaconazole and oxiconazole was determined using absorbance values at 570 nm and plotting for “[Inhibitor] vs. normalized response variable slope” in GraphPad Prism Version 10.2.3 (RRID: SCR 002798). A combination of antibiotics and compound, showing >70% viable cells, was considered not to affect cell viability (44).

### Murine skin infection model

#### Sample size calculation by power analysis

The hypothesis of the study was, “The combination of antibiotics with sertaconazole or oxiconazole yields a significantly greater reduction in multidrug-resistant *S. aureus* infection in comparison with antibiotics or drugs alone.” The sample size per group in the study was determined by power analysis using *a priori*: compute required given sample size, given *α*, power, and effect size by two-tailed *t*-test for independent means of two groups in G*Power software, version 3.1.9.6 (45). The following parameters were used for the calculation: *α* = 0.05, power (1*−β*) = 0.90, allocation ratio (*N*2/*N*1) = 1, and effect size (Cohen’s *d*) = 2.35. The effect size was determined using the means and standard deviations of the two independent groups as follows: mean of group 1 (average CFU/g in the infection group): 13, standard deviation (SD) of group 1: 1.5; mean of group 2 (average CFU/g for the drug-antibiotic combination): 9, SD of group 2: 1. Based on these values, the calculated sample size (*N*) was found to be five.

#### Animal selection

This study used female BALB/c mice selected for their beneficial behavioral and physiological characteristics. The immune response in female mice is observed to be more robust compared to that of male mice, which may be attributed to the effects of oestrogen on phagocytosis (46). Furthermore, females show enhanced resistance to *S. aureus* skin infections, displaying reduced severity and inflammation at the sites of infection (47). It is crucial to acknowledge that male mice frequently exhibit aggressive behavior, resulting in bite wounds and other skin injuries that may influence the results of the study focused on skin infections (48, 49).

#### Experimental procedure

A previously described method was followed (50) to determine the *in vivo* efficacy of antibiotics (norfloxacin, moxifloxacin, and cefotaxime), sertaconazole, and oxiconazole, either alone or in combination, in a murine skin infection model for Mu50. Based on G power analysis, a total of 45 female BALB/c mice (6-week-old) in 9 groups, with 5 mice per experimental group in a cage, were used in the study. Before the start of the experiment, the mice were anesthetized to shave the right flank region and allowed to recover. On day 1, the mice were administered 200 µL of Mu50 cells in PBS (Gibco, Catalogue number: 10010023) containing 1 × 10^8^ cells by subcutaneous injection in the shaved right flank region and were allowed to develop an infection for 2 days. From day 3 onward, treatment was administered daily in sterile PBS for 5 days. The administered treatments included sertaconazole (10 mg/kg) (51–54), oxiconazole (10 mg/kg) (55, 56), norfloxacin (100 mg/kg) (57, 58), moxifloxacin (100 mg/kg) (59, 60), and cefotaxime (15 mg/kg) (61–63), either alone or in combination. On day 8, the mice were sacrificed by CO_2_ asphyxiation, and infected skin tissues were collected for colony-forming units (CFU) plating. The tissue samples were homogenized in 1 mL of PBS, and the CFU was quantified through serial dilution of 100 µL tissue samples on Mannitol Salt Agar, followed by incubation at 37°C for 18 h. Every experimental setup included a control group receiving no infection. The statistical significance between various treatment groups was evaluated using one-way analysis of variance (ANOVA) with GraphPad Prism software Version 10.2.3 (RRID: SCR_002798). To ensure experimental consistency and comparability of results, the images representing control, infection, and single antibiotic treatment groups were used as internal controls in the combination therapy investigations, including either sertaconazole or oxiconazole.

#### Membrane potential assay

The effect of sertaconazole and oxiconazole on the membrane potential of *S. aureus* (ATCC25923 & Mu50) was evaluated using the Bacterial Membrane Potential Assay Kit, as previously mentioned (42). The kit uses a membrane-potential-sensitive dye, DiOC_2_, exhibiting red fluorescence in bacterial cells with unchanged or increased membrane potential. In contrast, it shows green fluorescence in depolarized cells (reduced membrane potential). Briefly, bacterial cells were cultured till OD_600_ ~ 0.3 and were given treatment for 1 h. The studied treatment groups included sertaconazole (4 µg/mL), oxiconazole (4 µg/mL), and CCCP (a known protonophore) (5 µM). Following 1 h of treatment, cells were centrifuged at 10,000 × *g* for 5 min, washed in PBS, and resuspended in PBS to an OD_600_ ~ 0.3. The cells were subsequently stained with 30 µM DiOC₂ and incubated for 30 min at room temperature in the dark. Fluorescence was ultimately assessed at an excitation wavelength of 485 nm and emission wavelengths of 530 nm (green) and 610 nm (red) in the CYTOFLEX flow cytometry machine (RRID: SCR_019627). The observations were recorded in logarithmic mode and analyzed with FlowJo software. Additionally, the red/green ratio was determined using the red and green intensities acquired during flow cytometry data acquisition to assess the size-independent effect.

#### Microbial cell viability assay

The effect of sertaconazole and oxiconazole on cellular ATP levels was assessed using the BacTiter-Glo kit (Promega, Catalog number: G8230) following the manufacturer’s protocol. Cells were cultured to an OD_600_ ~ 0.3 and treated with 4 µg/mL sertaconazole or oxiconazole for one hour at 37°C and 200 rpm. CCCP, a commonly used protonophore that diminishes cellular ATP levels, was used as a positive control. Following 1 h of treatment, cells were centrifuged at 5,000 rpm for 5 min and resuspended in an equal volume of PBS. Subsequently, 50 µL of resuspended cells was mixed with 50 µL of BacTiter Glo reagent, and the relative luminescence unit (RLU) and OD600_600_ were determined using the VictorNivo multimode plate reader. The RLU units were normalized with the OD_600_ value.

#### Ethidium bromide agar cartwheel assay

The level of efflux activity between ATCC25923 and Mu50 was evaluated by the EtBr agar cartwheel assay following the previously described method (64). Briefly, the ATCC25923 and Mu50 cells were cultured till OD_600_ ~ 0.6. The TSA agar plates with different concentrations of EtBr were prepared, and then the OD_600_ ~ 0.6 bacterial cultures were streaked onto the agar plates and incubated for 18 h at 37°C. Following incubation, the plates were observed under UV light for EtBr fluorescence, where higher EtBr fluorescence indicates lesser efflux pump activity and vice versa.

## RESULTS

### Efflux pump inhibitory activity of sertaconazole and oxiconazole against *S. aureus*

**Fig 1:**
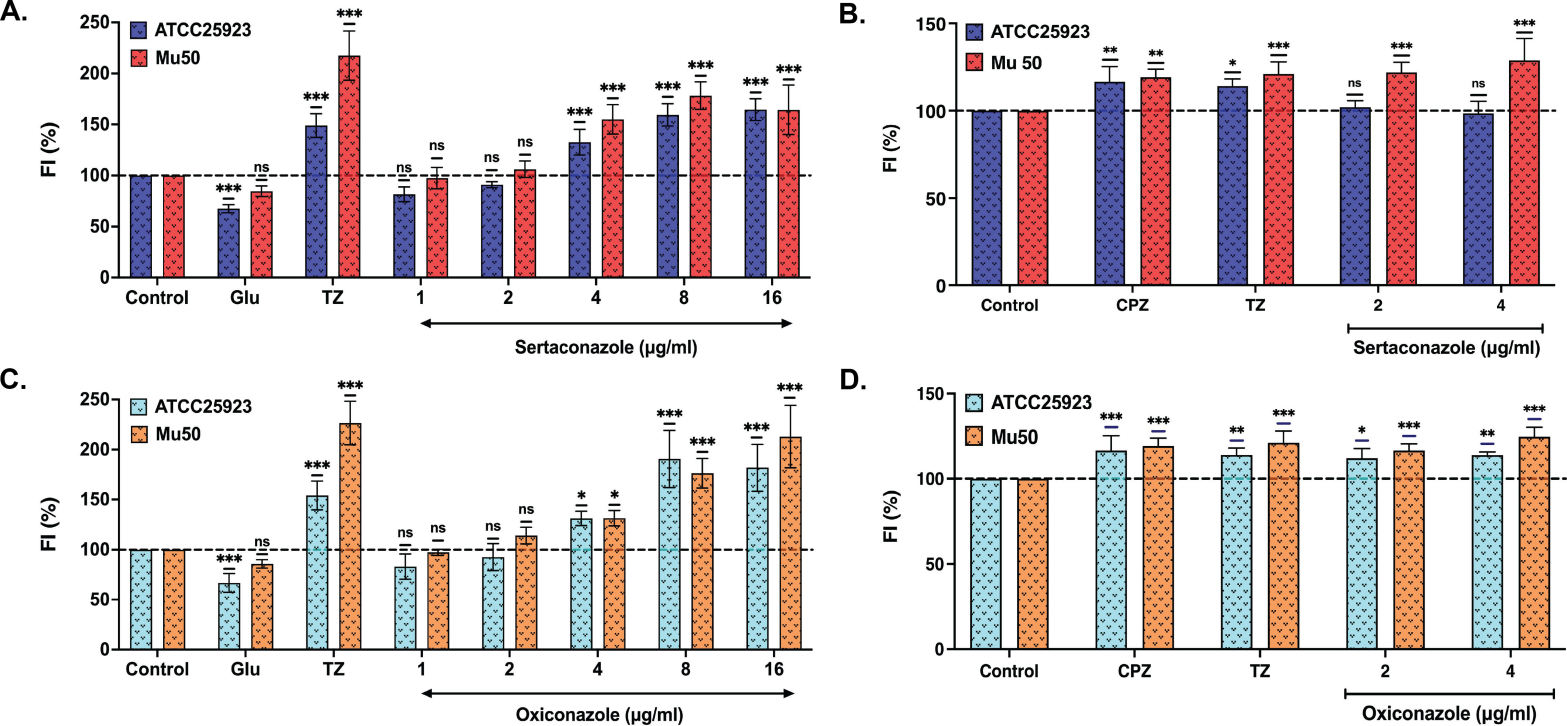
Sertaconazole and oxiconazole inhibit *S. aureus* efflux pumps *in vitro*. Using ethidium bromide (EtBr) accumulation and the EtBr efflux assay, the inhibition of the efflux pump was studied. Panels A and C show bar graphs that depict the concentration-dependent increase in relative fluorescence of EtBr in the presence of sertaconazole (Sert) and oxiconazole (Oxi), respectively. Thioridazine (TZ) is a known efflux pump inhibitor, used as a positive control at 16 µg/mL, while glucose (Glu), known to enhance active efflux, was used as a negative control at 0.4%. Panels B and D illustrate the decrease in EtBr efflux in the presence of sertaconazole and oxiconazole. Chlorpromazine (CPZ) and TZ, known EPIs, were used as positive controls at 16 µg/mL. The data reported are an average of three individual experiments, and the significance was determined using ordinary one-way ANOVA assuming Gaussian distribution, followed by multiple comparisons with Šidák correction, and is reported as ^∗∗∗^*P* < 0.001, ^∗∗^*P* < 0.002, ^∗^*P* < 0.033, ^ns^*P* > 0.05.

This study assessed the efflux pump inhibitory activity of sertaconazole and oxiconazole using EtBr accumulation and EtBr efflux assays in drug-sensitive (ATCC25923) and drug-resistant (Mu50) *S. aureus*. EtBr serves as a substrate for most efflux pumps in *S. aureus,* and both assays quantify the EtBr accumulation in relation to efflux pump activity. In the accumulation assay, increased EtBr accumulation correlates with the inhibition of efflux pumps, whereas in efflux assays, higher EtBr accumulation suggests a reduction in the glucose-mediated efflux rates. In our study, both sertaconazole (Fig. 1A) and oxiconazole (Fig. 1C) treatments demonstrated an increased EtBr accumulation in both strains, indicating a concentration-dependent inhibition of efflux pumps. The efflux assay demonstrated that sertaconazole (Fig. 1B) exhibited little to no change in EtBr efflux in ATCC25923, and oxiconazole (Fig. 1D) demonstrated decreased EtBr efflux. In contrast, both drugs showed decreased EtBr efflux in Mu50 (Fig. 1B and D).

### Antibacterial properties of sertaconazole and oxiconazole

The potential antibacterial effect of sertaconazole and oxiconazole was examined using susceptibility testing in *S. aureus* (ATCC25923 & Mu50). The results showed that sertaconazole and oxiconazole inhibited the growth of both ATCC25923 and Mu50, with MICs of 4 µg/mL and 8 µg/mL, respectively (Table 1). Although both compounds inhibited bacterial growth, they did not show bacterial killing activity in *S. aureus*. Our results showed a high value of MBC > 128 µg/mL for both the sensitive and resistant strains of *S. aureus* (Table 1). Similarly, we also determined the antibacterial effects of three other antibiotics (norfloxacin, cefotaxime, and moxifloxacin). These three antibiotics are the major substrates for the *S. aureus* efflux pumps. The MIC and MBC values are shown in Table 1. Our results showed that ATCC25923 was sensitive to cefotaxime, norfloxacin, and moxifloxacin with respective MICs of 0.5, 2, and 0.064 µg/mL, whereas Mu50 was resistant to all the above antibiotics with MICs of 256, 256, and 32 µg/mL, respectively.

**TABLE 1:** Sertaconazole and oxiconazole susceptibility and synergistic (FIC) evaluation with antibiotic combinations in ATCC25923 and MRSA Mu50 (ATCC700699)

SI no.	Combination	Compound/antibiotic	MIC alone (A)	MBC	MIC in combination (B)	B/A	FIC
ATCC25923							
1	Sertaconazole + cefotaxime	Sertaconazole	4	>128	0.5	0.125	0.25
		Cefotaxime	0.5	2	0.062	0.124	
2	Sertaconazole + norfloxacin	Sertaconazole	4	>128	0.5	0.125	0.38
		Norfloxacin	2	16	0.5	0.250	
3	Sertaconazole + moxifloxacin	Sertaconazole	4	>128	0.5	0.125	1.13
		Moxifloxacin	0.064	2	0.064	1.000	
4	Oxiconazole + cefotaxime	Oxiconazole	8	>128	1	0.125	0.23
		Cefotaxime	0.5	2	0.062	0.124	
5	Oxiconazole + norfloxacin	Oxiconazole	8	>128	1	0.125	0.38
		Norfloxacin	2	16	0.5	0.250	
6	Oxiconazole + moxifloxacin	Oxiconazole	8	>128	1	0.125	1.13
		Moxifloxacin	0.064	2	0.064	1.000	
Mu50							
7	Sertaconazole + cefotaxime	Sertaconazole	4	>128	0.5	0.125	0.19
		Cefotaxime	256	>128	16	0.063	
8	Sertaconazole + norfloxacin	Sertaconazole	4	>128	0.5	0.125	0.38
		Norfloxacin	256	>128	64	0.250	
9	Sertaconazole + moxifloxacin	Sertaconazole	4	>128	0.5	0.125	0.25
		Moxifloxacin	32	64	4	0.125	
10	Oxiconazole + cefotaxime	Oxiconazole	8	>128	1	0.125	0.25
		Cefotaxime	256	>128	32	0.125	
11	Oxiconazole + norfloxacin	Oxiconazole	8	>128	1	0.125	0.38
		Norfloxacin	256	>128	64	0.250	
12	Oxiconazole + moxifloxacin	Oxiconazole	8	>128	1	0.125	0.25
		Moxifloxacin	32	64	4	0.125	

**Fig 2:**
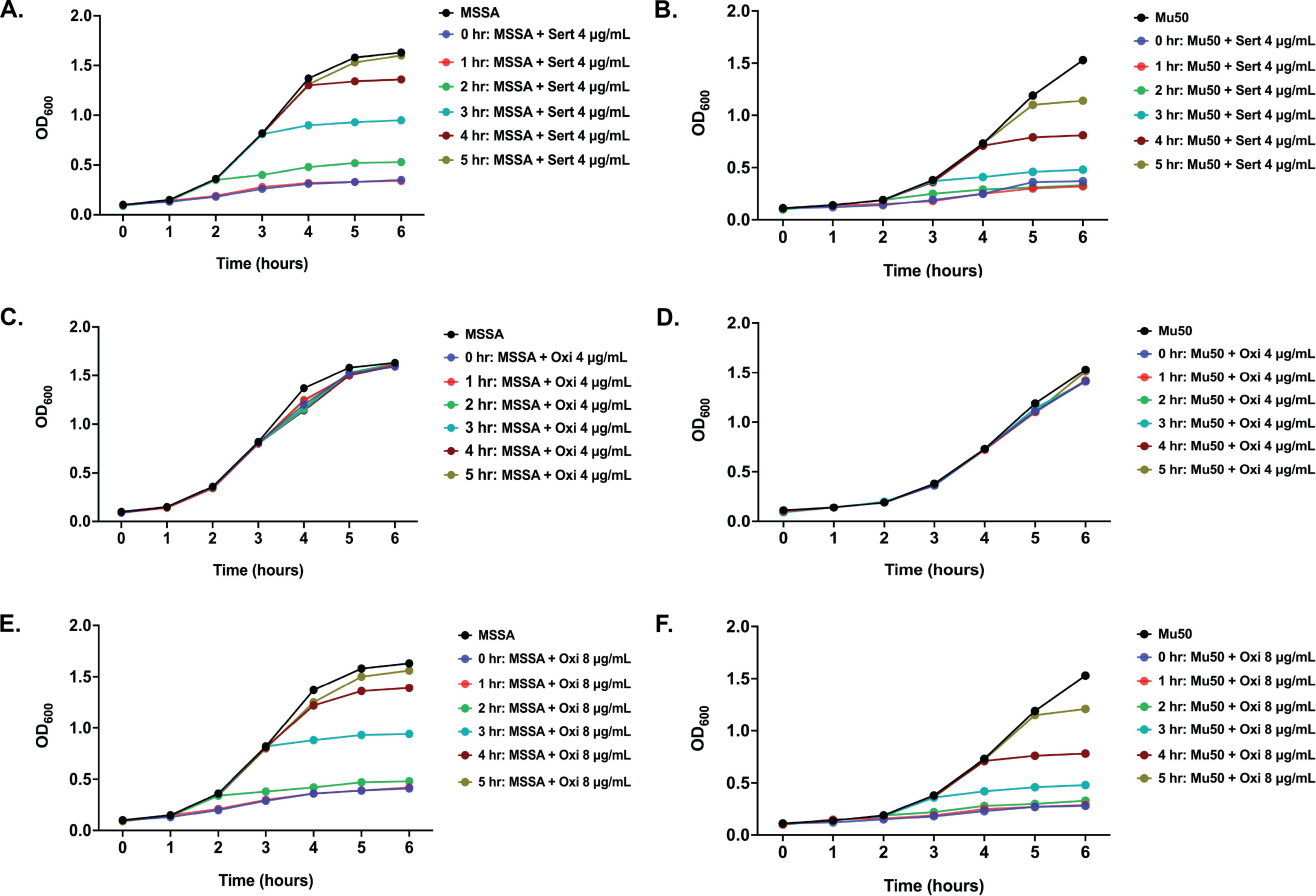
Effect of sertaconazole and oxiconazole on *S. aureus* growth. A bacterial growth curve assay was performed to investigate the effect of sertaconazole (Sert) and oxiconazole (Oxi) on the growth of *S. aureus* (ATCC25923 and Mu50). ATCC25923 and Mu50 were grown in tryptic soy broth, with treatments given hourly using Sert at a concentration of 4 µg/mL (MIC) or Oxi at concentrations of either 4 µg/mL (1/2 MIC) or 8 µg/mL (MIC). The growth of ATCC25923 and Mu50 was monitored for 6 h by measuring the optical density (OD) at 600 nm at hourly intervals. Panels A and B illustrate that Sert at 4 µg/mL effectively inhibits the growth of ATCC25923 and Mu50, respectively, suggesting a bacteriostatic effect. Panels C and D illustrate that Oxi at 4 µg/mL does not affect the growth of ATCC25923 and Mu50, respectively. At a concentration of 8 µg/mL, Oxi demonstrates bacteriostatic effects by markedly suppressing the growth of ATCC25923 (panel E) and Mu50 (panel F).

In our study, sertaconazole is used at its MIC concentration (4 µg/mL); oxiconazole is used at its half-MIC concentration (4 µg/mL). To assess the effects of sertaconazole and oxiconazole on bacterial growth and to examine the bacteriostatic effect of both drugs at the MIC concentration, we performed a bacterial growth curve (Fig. 2). The growth curve analysis indicated that at the MIC concentration, both drugs inhibited *S. aureus* growth. However, the OD_600_ values did not decline upon the addition of the drugs, indicating that both sertaconazole (Fig. 2A and B) and oxiconazole (Fig. 2E and F) are bacteriostatic in nature. The analysis also showed that oxiconazole at half-MIC concentration did not impact bacterial growth (Fig. 2C and D). The susceptibility and growth curve analysis findings indicated that at the used concentration, sertaconazole inhibited bacterial growth, and oxiconazole did not affect bacterial growth.

### Sertaconazole and oxiconazole synergistically enhanced the antibacterial effectiveness of existing antibiotics

**Fig 3:**
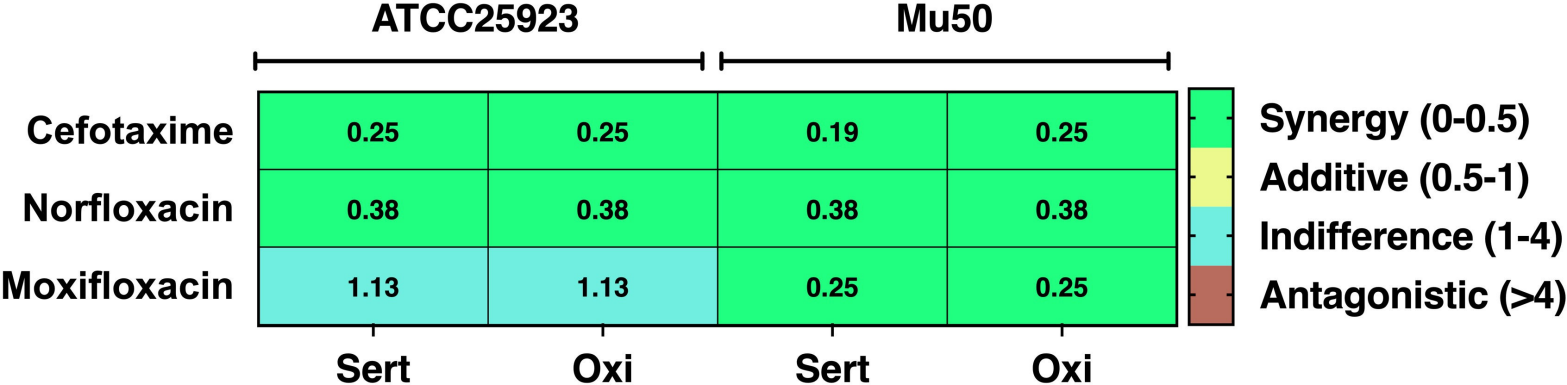
Oxiconazole and sertaconazole act in synergy with antibiotics. Using the checkerboard synergy assay, the combinatorial effects of sertaconazole and oxiconazole with antibiotics were studied. The figure illustrates a heat map depicting the *in vitro* synergistic effects of different concentrations of sertaconazole (Sert) and oxiconazole (Oxi) in combination with norfloxacin, moxifloxacin, and cefotaxime against ATCC25923 and Mu50.

A checkerboard synergy assay was used to investigate the possible effects of sertaconazole and oxiconazole in combination with cefotaxime, norfloxacin, or moxifloxacin on enhancing their efficacy against *S. aureus* (ATCC25923 & Mu50). The MICs of antibiotics (norfloxacin, cefotaxime, and moxifloxacin) and EPIs (sertaconazole and oxiconazole), alone or in combination, were determined against *S. aureus*. The FIC values were calculated as specified in the method section and used to determine possible combinatorial effects. The FIC values < 0.5 indicate synergistic effects, whereas values between 0.5 > FIC < 1 show additive effects, and higher values indicate indifferent (1 > FIC < 4) or antagonistic effects (FIC > 4) (Table 1). In ATCC25923, both EPIs demonstrated to be synergistic (FIC < 0.5) with norfloxacin and cefotaxime, while they showed indifference in combination with moxifloxacin (FIC 1.13) (Fig. 3; Table 1). In Mu50, both EPIs demonstrated synergy with all three antibiotics (Fig. 3; Table 1). The indifference effect of moxifloxacin in combination with sertaconazole or oxiconazole may result from reduced efflux pump activity in ATCC25923, as illustrated in Fig. S2, where increased EtBr fluorescence signifies diminished EtBr efflux and enhanced EtBr accumulation, and vice versa.

### Sertaconazole and oxiconazole did not affect bacterial membrane integrity

**Fig 4:**
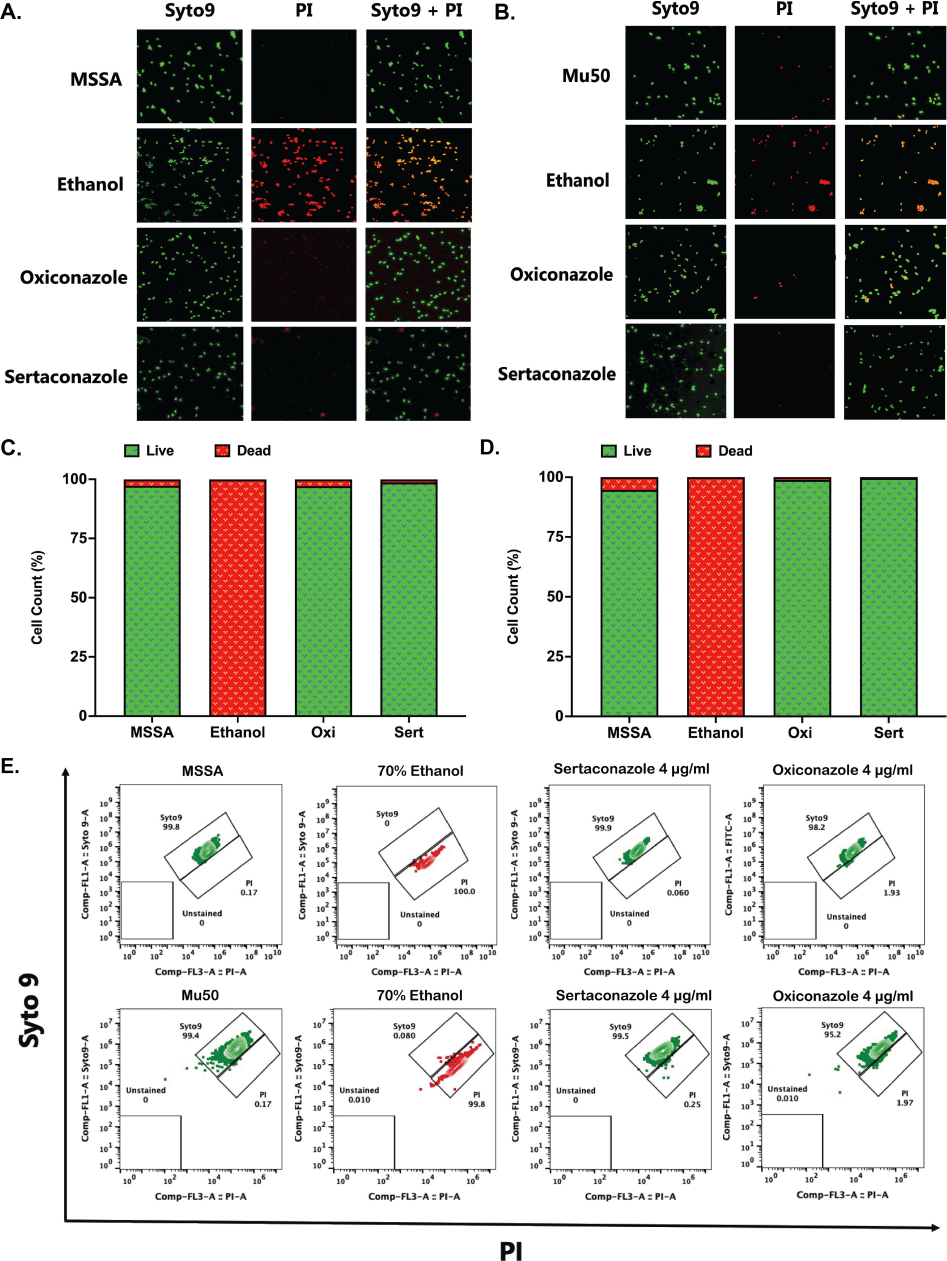
Effect of sertaconazole and oxiconazole on the bacterial membrane. Using a live-dead assay, the effects of sertaconazole and oxiconazole were studied. Panels A and B demonstrate that neither sertaconazole nor oxiconazole affects the bacterial membrane, with over 98% of viable cells at 4 µg/mL for ATCC25923 and Mu50, respectively. Panels C and D show the bar plot illustrating the percentage of live and dead cell counts in the presence of 4 µg/mL of sertaconazole (Sert) and oxiconazole (Oxi) in both ATCC25923 and Mu50 strains, respectively. The cell number was quantified from microscopic images utilizing ImageJ software. Panel E represents flow cytometry analysis indicating that neither sertaconazole nor oxiconazole affects bacterial membranes, with over 98% of viable cells at 4 µg/mL for ATCC25923 and Mu50, respectively. The entire study used 70% ethanol-treated cells as a dead cell control (stained with PI), whereas untreated cells (ATCC25923 & Mu50) were used as the live cell control (stained with Syto9).

Sertaconazole and oxiconazole are known to disrupt membrane integrity as antifungal agents (65). Thus, to assess if sertaconazole and oxiconazole have similar effects on the membrane integrity of *S. aureus*, we evaluated the cell viability using a live-dead assay (Syto9-PI staining) by both flow cytometry and fluorescence microscopy. Untreated cells (ATCC25923 and Mu50) served as the live cell controls and showed a whole green population, whereas cells treated with 70% ethanol were used as the dead cell controls and showed a whole red population. The fluorescence microscopy analysis (Fig. 4A and B) showed that *S. aureus,* when treated for 1 h with sertaconazole and oxiconazole at 4 µg/mL, had no effects on the bacterial membrane. The cell behavior was similar to that of untreated cells, with most of the cell population being live and intact. The percentage of viable cells, determined using ImageJ analysis of microscopic images, showed >98% viable cells in both sertaconazole and oxiconazole treatments (Fig. 4C and D). Additionally, we assessed the percentage of viable cells using flow cytometry (Fig. 4E), which also demonstrated a similar pattern, showing >98% viable cells upon sertaconazole and oxiconazole treatments. The results indicated that sertaconazole and oxiconazole treatment did not affect bacterial membrane integrity.

### Sertaconazole and oxiconazole showed lower toxicity toward mammalian cells

To determine the effect of sertaconazole and oxiconazole on mammalian cell viability, we performed an MTT assay using A549 lung cancer adherent cells and HEK293T epithelial cells. The cell viability indicated that most of the cell population was viable at 8 µM (4 µg/mL) for both sertaconazole and oxiconazole (Table 2), the concentration used in our experiment. The 50% cytotoxic concentration (CC_50_) was determined to be 23.5 ± 5.3 µM and 24.5 ± 2.9 µM for sertaconazole and oxiconazole, respectively, in A549 cells (Table 2). For HEK293T, the CC_50_ was calculated to be 9.9 ± 2.2 µM and 17.9 ± 1.1 µM for sertaconazole and oxiconazole, respectively (Table 2). Additionally, we assessed the potential effects of combining sertaconazole or oxiconazole with antibiotics (norfloxacin, moxifloxacin, and cefotaxime) on the viability of A549 mammalian cells. Our results showed that combining 10 µM antibiotics with 10 µM of either sertaconazole or oxiconazole had minimal effects on cell viability (Fig. S1). The cell viability analysis suggested that at the experimental concentrations, both sertaconazole and oxiconazole have minimal impact on mammalian cell viability, individually, or in combination with antibiotics.

**TABLE 2:** Evaluation of 50% cytotoxicity (CC_50_) of sertaconazole and oxiconazole in mammalian cells

SI no.	Drug	CC_50_ (μM) for:	
		A549	HEK293T
1	Sertaconazole	23.5 ± 5.3	9.9 ± 2.2
2	Oxiconazole	24.5 ± 2.9	17.9 ± 1.1

### Sertaconazole and oxiconazole enhanced the efficacy of the antibiotics against *S. aureus* in a murine skin infection model

**Fig 5:**
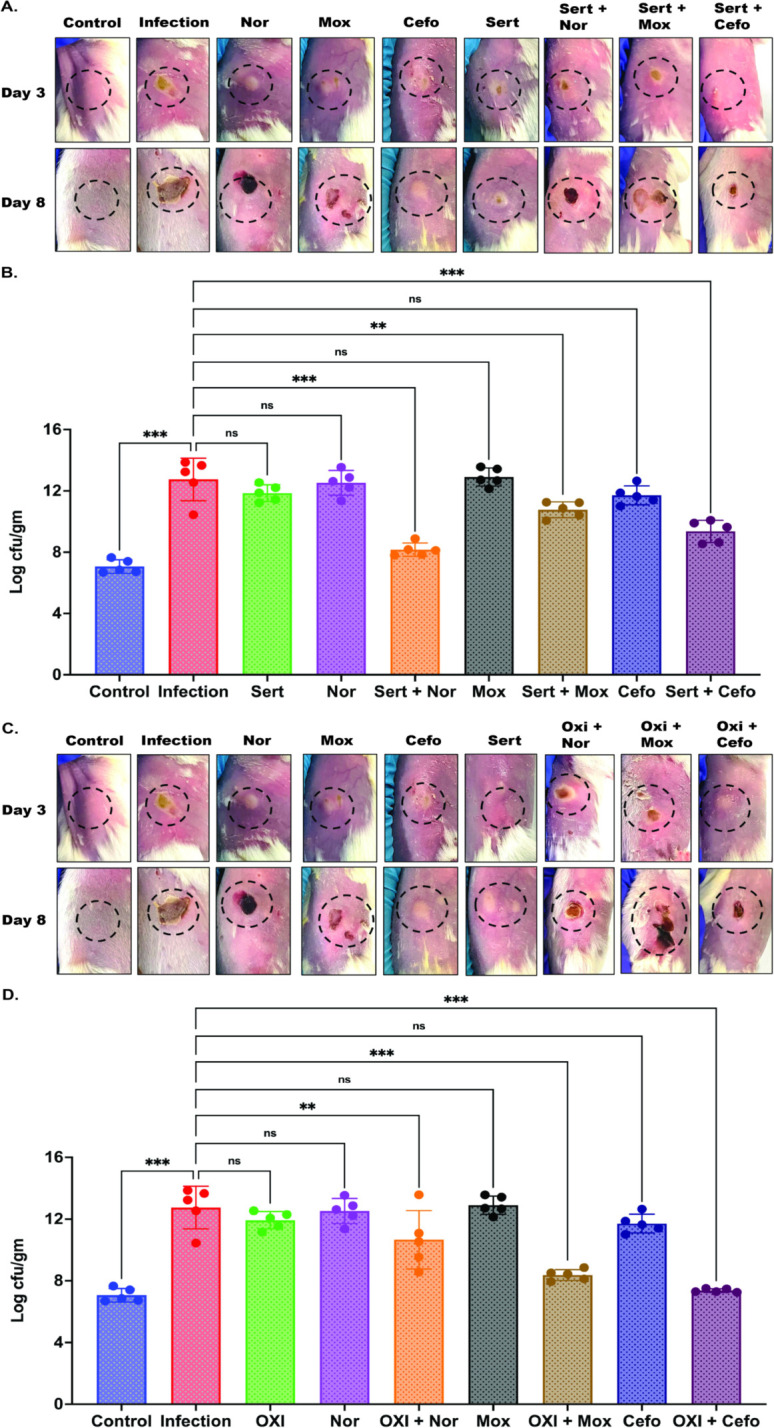
*In vivo* efficacy of antibiotics with sertaconazole or oxiconazole. The effectiveness of antibiotics in combination with sertaconazole (Sert) or oxiconazole (Oxi) against *S. aureus* Mu50 were assessed in a mouse model. Panels A and C show images of the infected and treated skin areas on day 1 of treatment (day 3) and the day of sacrifice (day 8). Panels B and D illustrate the bacterial load in the skin tissues of uninfected mice (Control), Mu50-infected mice (Infection), and those treated with antibiotics, sertaconazole, and oxiconazole on day 8. The bacterial load was assessed using the viable cell count method and represented as log10 CFU/g. Nor, norfloxacin; Mox, moxifloxacin; Cefo, cefotaxime. The significance was determined using ordinary one-way ANOVA assuming Gaussian distribution, followed by multiple comparisons with Šidák correction, and is reported as ^∗∗∗^*P* < 0.001, ^∗∗^*P* < 0.002, ^∗^*P* < 0.033, ^ns^*P* > 0.05. The control, infection, and individual antibiotic treatment groups (Nor, Mox, and Cefo) are common for both sertaconazole and oxiconazole combination treatments; therefore, the images representing these groups are the same in panels A & C and B & D.

Our *in vitro* synergistic analysis demonstrated an increased efficacy of antibiotics against ATCC25923 and Mu50 when combined with sertaconazole or oxiconazole. We evaluated the combinatorial effects in a murine skin infection model to determine if the combinations can improve antibiotic efficiency *in vivo*. Briefly, Mu50 was subcutaneously administered in the right flank region of female Balb/C mice and incubated for 2 days to develop the infection. On day 3, the treatment of sertaconazole (10 mg/kg), oxiconazole (10 mg/kg), norfloxacin (100 mg/kg), moxifloxacin (100 mg/kg), and cefotaxime (15 mg/kg) were administered daily for 5 days, either individually or in combination. On day 8, mice were sacrificed, and the infected skin tissue (Fig. S3A) was collected for viable cell counting (CFU plating). The results showed that individual administration of sertaconazole, oxiconazole, and antibiotics was ineffective in reducing Mu50 infection, showing ≤1 log_10_ reduction in the CFU count (Fig. 5B and D). In contrast, combining sertaconazole or oxiconazole with antibiotics showed higher efficacy than the individual treatments (Fig. 5A through D). Sertaconazole showed the highest efficacy when combined with norfloxacin (4.4 log_10_ reduction in CFU count) or cefotaxime (3.4 log_10_ reduction in CFU count) (Fig. 5B). Additionally, in combination with moxifloxacin, sertaconazole showed a 2 log_10_ reduction in viable cell count (Fig. 5B). Meanwhile, oxiconazole showed the highest efficacy when combined with cefotaxime (5.4 log_10_ reduction) and moxifloxacin (4.6 log_10_ reduction) (Fig. 5D). Similarly, in combination with norfloxacin, oxiconazole showed a 2 log_10_ reduction in viable cell count (Fig. 5D). The weight of the mice remained unchanged during the entire study (Fig. S3B). The mouse study showed that sertaconazole and oxiconazole enhanced the efficacy of the antibiotics similar to that observed for *in vitro* studies (Fig. 3).

### Sertaconazole and oxiconazole reduced membrane potential and inhibited ATP production

**Fig 6:**
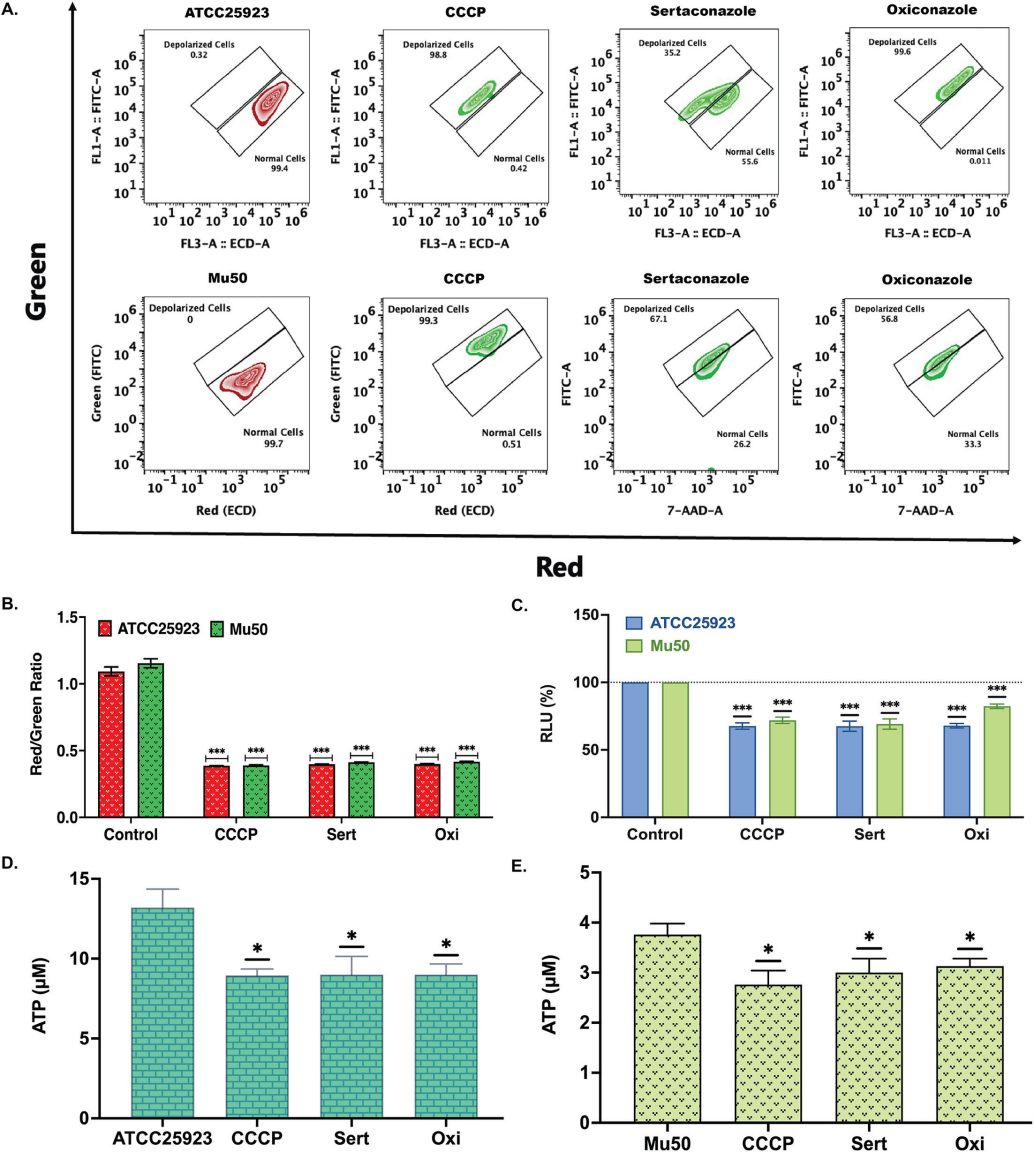
Sertaconazole and oxiconazole induce depolarization and decrease ATP levels in *S. aureus*. The membrane potential of ATCC25923 and Mu50 was assessed in the presence of sertaconazole (Sert) and oxiconazole (Oxi) at a concentration of 4 µg/mL using DiOC_2_ labeling and flow cytometry. The protonophore CCCP is a positive control in both experiments due to its known ability to depolarize bacteria and reduce ATP production. Untreated cells (ATCC25923 & Mu50) serve as controls in both experiments, demonstrating no alteration in membrane potential or ATP production. In Panel A, the red color indicates no alteration in membrane potential, while the green color indicates membrane depolarization. Panel A demonstrates that sertaconazole and oxiconazole result in depolarization in both ATCC25923 and Mu50, exhibiting a cellular pattern similar to that of CCCP-treated cells in flow cytometry. Panel B illustrates the relative red-to-green fluorescence intensity ratio in cells treated with sertaconazole, oxiconazole, and CCCP. Panel C demonstrates that treatment with 4 µg/mL of either sertaconazole or oxiconazole has effects similar to CCCP, showing a reduction in relative luminescence units (RLU) compared to the control, indicating reduced ATP production in both ATCC25923 and Mu50 cells. Panel D illustrates the relative ATP production of ATCC25923 cells following treatment with either 4 µg/mL sertaconazole or oxiconazole. Panel E illustrates the relative ATP production in Mu50 cells following treatment with either 4 µg/mL sertaconazole or oxiconazole. The relative ATP production was measured using the ATP standard curve (Fig. S4). The data reported are from three individual experiments, and the significance was determined using ordinary two-way ANOVA assuming Gaussian distribution, followed by multiple comparisons with Šidák correction, and is reported as ^∗∗∗^*P* < 0.001, ^∗∗^*P* < 0.002, ^∗^*P* < 0.033, ^ns^*P* >0.05.

The primary energy sources for efflux pump function in *S. aureus* are proton motive force (PMF), which includes both alterations in transmembrane proton gradient, also known as the electrochemical gradient (ΔpH), and electric or membrane potential (Δ*Ψ*) (10, 14–19, 26). Thus, to determine whether sertaconazole and oxiconazole inhibit the efflux pump activity of *S. aureus* (ATCC25923 & Mu50) by altering membrane potential and ATP production, we investigated their effects on bacterial membrane potential and ATP production. The effect on bacterial membrane potential was evaluated by flow cytometry using the BacLight bacterial membrane potential kit (Invitrogen), which utilizes DiOC_2_, a membrane potential-sensitive dye. DiOC_2_ shows red fluorescence in cells with unaltered membrane potential and green fluorescence in depolarized cells (decreased membrane potential). CCCP, a known protonophore that depolarizes bacterial membranes, was a positive control for our experiment (20). The flow cytometry results (Fig. 6A) showed that both sertaconazole and oxiconazole decreased the membrane potential of *S. aureus*. Most of the *S. aureus* treated with sertaconazole or oxiconazole showed higher green intensity than red intensity and showed a cellular pattern similar to that of CCCP-treated cells (Fig. 6A). It is previously known that bacterial size can influence the outcome of the potential study (66). Thus, we also checked the red-to-green intensity ratio (Fig. 6B) to see the effect of sertaconazole and oxiconazole on bacterial membrane potential and the dependence on bacterial size. Both sertaconazole and oxiconazole showed a reduced red/green ratio similar to that seen with CCCP-treated cells in both ATCC25923 and Mu50 (Fig. 6B). This further supported the reduction of the membrane potential of *S. aureus* by both sertaconazole and oxiconazole, which is independent of the cell size.

Studies have shown that a reduction in the membrane potential alters the proton motive force, leading to the reduction in ATP production in bacteria (20, 66). To find out if treatment with sertaconazole or oxiconazole could reduce ATP production, we determined the ATP production by assessing bacterial cell viability using the BacTiter-Glo Microbial Cell Viability Assay kit (Promega), which determines viable bacterial cells by measuring total ATP production; a higher number of viable cells will indicate increased ATP production and vice versa. As CCCP is known to reduce membrane potential and reduce ATP production, we used CCCP as a positive control to observe the reduction in ATP production (20). Our results showed that both sertaconazole and oxiconazole reduced bacterial ATP production, similar to CCCP in ATCC25923 and Mu50 (Fig. 6C). Additionally, we quantified the ATP production of ATCC25923 and Mu50 at a concentration of 4 µg/mL of either sertaconazole or oxiconazole, utilizing the ATP standard curve (Fig. S4). The analysis of ATCC25923 indicated that untreated cells (ATCC25923) generated 13.2 ± 1.1 µM ATP (Fig. 6D). In contrast, cells treated with sertaconazole and oxiconazole showed decreased ATP production, yielding 9 ± 1.1 µM and 9 ± 0.61 µM ATP, respectively, displaying an identical response to CCCP (8.9 ± 0.4 µM) (Fig. 6D). In Mu50, the study indicated that untreated cells (Mu50) generated 3.8 ± 0.2 µM ATP (Fig. 6E). In contrast, cells treated with sertaconazole and oxiconazole showed reduced ATP production, yielding 3 ± 0.2 µM and 3.1 ± 0.15 µM ATP, respectively, demonstrating an equivalent response to CCCP (2.8 ± 0.28 µM) (Fig. 6E). The analysis of bacterial membrane potential and ATP production showed that sertaconazole and oxiconazole reduce *S. aureus* membrane potential, resulting in the disruption of PMF, which leads to reduced ATP production. In *S. aureus,* PMF and ATP serve as the energy sources for efflux pump activity, and the results showed that sertaconazole and oxiconazole disrupt PMF and reduce ATP production, suggesting that both drugs inhibit efflux pumps in *S. aureus* by hampering the energy required for the efflux pump activity.

## DISCUSSION

Antibiotic resistance seriously compromises public health worldwide. This is particularly true for many drug-resistant bacteria, such as *S. aureus*. Even with the availability of potent antimicrobials, the mortality rate associated with *S. aureus* bacteremia continues to be alarmingly high, ranging from 20% to 40% (67). Efflux pumps are essential for multidrug resistance development in *S. aureus*. They systematically eliminate a range of antibiotics and other antimicrobials from the bacterial cells (10). They also play crucial roles in removing endogenous metabolites, releasing virulence factors, and during cellular responses to stress (10, 68). In *S. aureus*, several multidrug efflux pumps have been identified, including NorA, NorB, AbcA, and MepA, which remove a range of antibiotics and dyes, such as fluoroquinolones, cefotaxime, and ethidium bromide (10, 13). Thus, inhibition of these pumps is a potential approach for restoring the effectiveness of antibiotics against multidrug-resistant *S. aureus*.

This study demonstrates that sertaconazole and oxiconazole, both FDA-approved antifungal agents, serve as effective EPIs against multidrug-resistant *S. aureus*. Both drugs significantly reduced the function of efflux pumps in the drug-sensitive ATCC25923 and the multidrug-resistant Mu50 strains. Both drugs resulted in a significant increase in EtBr accumulation, which serves as a standard assay for assessing efflux pump activity, thereby demonstrating their effectiveness in inhibiting efflux pumps in *S. aureus*. Sertaconazole demonstrated inhibition of efflux pumps (Fig. 1A) while maintaining the efflux rate (Fig. 1B). In contrast, oxiconazole not only inhibited efflux pumps (Fig. 1C) but also decreased the efflux rate (Fig. 1D). Both drugs demonstrated bacteriostatic activity (Fig. 2) with no impact on bacterial membrane integrity or growth (Fig. 2 and 4).

An essential characteristic of an ideal EPI is its ability to enhance the effectiveness of existing antibiotics while maintaining low toxicity levels for mammalian cells (25, 26). The findings of our study indicated that sertaconazole and oxiconazole improved the antibacterial efficacy of norfloxacin and cefotaxime against ATCC25923 and Mu50 (Fig. 3; Table 1). Additionally, both drugs improved the effectiveness of moxifloxacin against Mu50, showing no variation in efficacy against ATCC25923 (Fig. 3; Table 1). This could be attributed to the differential expression of efflux pumps between the two strains, with ATCC25923 having fewer active efflux pumps (Fig. S2). Furthermore, the cytotoxicity of both drugs was assessed in mammalian cells (A549 and HEK293T), revealing that both exhibited low cytotoxicity (Table 2), thus presenting them as safe for potential combination therapies. Additionally, the combination of these drugs with antibiotics demonstrated minimal cytotoxicity toward mammalian cells (Fig. S1), strengthening their potential for therapeutic application.

*In vivo* studies using a murine skin infection model of *S. aureus* infection provided additional confirmation of the improved antibacterial efficacy of antibiotics when combined with sertaconazole or oxiconazole. The combination treatment demonstrated a significant reduction in bacterial burden compared to antibiotics or drugs alone. Sertaconazole demonstrated a higher level of synergy with norfloxacin (Fig. 5B), whereas oxiconazole increased the effectiveness of moxifloxacin and cefotaxime in combination (Fig. 5D). The combination of sertaconazole or oxiconazole with antibiotics led to a significant reduction in *S. aureus* infection, highlighting their potential as adjuvants to the current antibiotic treatments in the fight against multidrug-resistant *S. aureus* infections.

**Fig 7:**
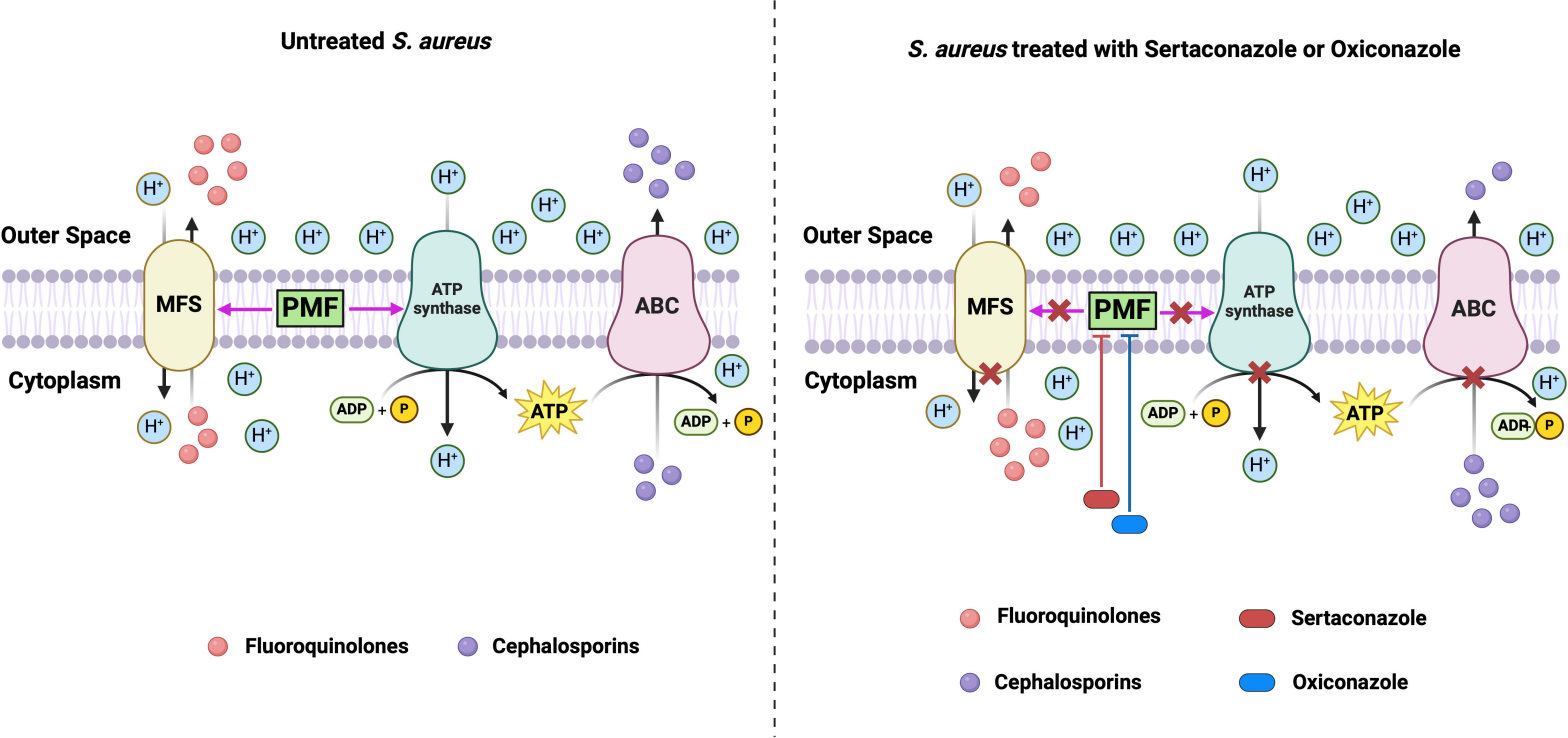
Mechanism of efflux pump inhibition by sertaconazole or oxiconazole. In *S. aureus*, proton motive force (PMF) provides the energy essential for the functioning of MFS-type efflux pumps, which mostly efflux fluoroquinolones. Simultaneously, the ATP synthase in the bacterial membrane uses the PMF to produce ATP, which is then used by an ABC-type efflux pump to efflux cephalosporins. Sertaconazole or oxiconazole treatment disrupts the PMF. Disruption of PMF leads to inhibition of MFS-type efflux pumps, resulting in decreased efflux and increased cytoplasmic accumulation of fluoroquinolone. Furthermore, PMF disruption also leads to inhibition of ATP synthesis, which results in inhibition of ABC-type efflux pumps, resulting in decreased efflux and increased cytoplasmic accumulation of cephalosporins. The figure was created in BioRender.com.

The mechanism of action of sertaconazole and oxiconazole as EPIs was elucidated by examining their effect on the PMF of *S. aureus*. Both drugs caused depolarization of the bacterial membrane (Fig. 6A and B), resulting in a reduction in electric or membrane potential (Δ*Ψ*) and an increase in the electrochemical gradient (ΔpH), hence disrupting cellular ATP production (Fig. 6C through E). By interrupting the PMF and ATP production, both drugs reduced the activity of MFS-type and ABC-type efflux pumps, resulting in enhanced intracellular antibiotic retention and higher antibiotic effectiveness in combination with these drugs (Fig. 7).

Evaluating sertaconazole and oxiconazole alongside previously identified EPIs, including both plant-derived and synthetic EPIs, offers more understanding of their potential therapeutic efficacy. For example, plant-derived EPIs such as berberine, curcumin, and resveratrol have shown modest EPI efficacy against *S. aureus* (69–71). Although these compounds have potential as EPIs, their limited bioavailability and high toxicity restrict their practical application (26). In contrast, the FDA-approved sertaconazole and oxiconazole, as topical antifungal drugs, provide enhanced safety due to minimal systemic exposure, therefore mitigating the risks associated with poor bioavailability and high toxicity (72–78). Sertaconazole and oxiconazole also outperform several synthetic and peptide-based EPIs. Synthetic EPIs such as phenylalanine-arginine-β-naphthylamide (PAβN), verapamil, and carbonyl cyanide m-chlorophenylhydrazone (CCCP) exhibit significant efflux inhibition but possess high toxicity and low solubility (26). Therefore, with FDA approval, strong efflux inhibition activity, and the capacity to increase the efficacy of antibiotics in combination against multidrug-resistant *S. aureus* while exhibiting minimal toxicity to mammalian cells, both sertaconazole and oxiconazole represent strong viable options for clinical development as EPIs for tackling antimicrobial resistance in *S. aureus*.

Although the results are promising, there are certain limitations to consider. First, our investigation focused on a limited number of *S. aureus* strains; further research should assess the effectiveness of sertaconazole and oxiconazole against a wider array of clinical isolates to ascertain the generalizability of the results. Second, while our *in vitro* and *in vivo* investigations demonstrated enhanced antibacterial efficacy of antibiotics when combined with sertaconazole or oxiconazole against multidrug-resistant *S. aureus*, additional pharmacokinetic and toxicological studies are necessary to evaluate clinical applicability, especially in combination with the antibiotics. Additionally, further research is needed to elucidate the molecular mechanisms through which these drugs inhibit efflux pumps and modulate PMF, thereby improving their potential in combination therapies.

In summary, sertaconazole and oxiconazole are promising candidates for repurposing as EPIs in the treatment of multidrug-resistant *S. aureus*. The established safety profiles, low toxicity, and capacity to enhance the efficacy of antibiotics render both drugs superior compared to various synthetic and plant-derived EPIs. More research should be conducted to optimize their pharmacokinetic properties and their efficacy when used with a broad range of antibiotics to counteract the rising threat of multidrug-resistant *S. aureus*.

## Data Availability

Any data included in the main text or supplemental material that demonstrate the study’s conclusions will be accessible upon reasonable request to the corresponding author (tkbeuria@ils.res.in). Requests for reanalysis of the data contained in this article for the purposes of replicating results will be granted. Requests for analyses beyond the scope of this publication will be evaluated by the corresponding author to evaluate the scientific and ethical appropriateness of the proposed data use.

## SUPPLEMENTAL MATERIAL

Supplemental material aac.00320-25-s0001.docx Fig. S1 to S4.

ASM does not own the copyrights to Supplemental Material that may be linked to, or accessed through, an article. The authors have granted ASM a non-exclusive, world-wide license to publish the Supplemental Material files. Please contact the corresponding author directly for reuse.
