# Supplementary material for: Inhibition of efflux pumps by FDA-approved drugs oxiconazole and sertaconazole restores antibiotic susceptibility in multidrug-resistant S. aureus
Source: Antimicrob Agents Chemother. 2025 Aug 4;69(9):e00320-25. doi: 10.1128/aac.00320-25 (PMC12406663; doi:10.1128/aac.00320-25)
Supplement: Supplemental material — Fig. S1 to S4. [file aac.00320-25-s0001.docx]

**Supplementary**

**
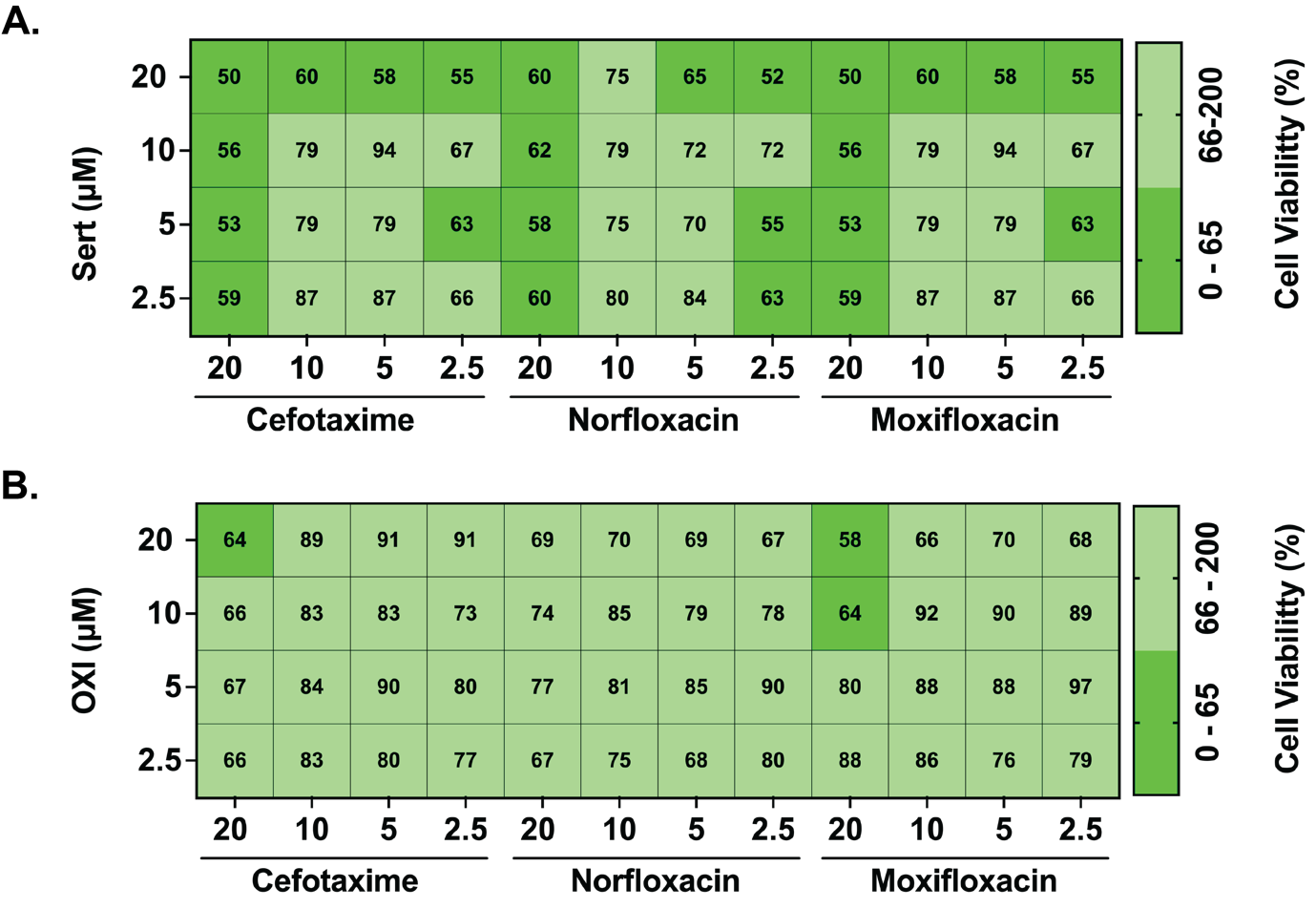
**

***Figure S1: Effect of sertaconazole or oxiconazole combination with antibiotics on A549 cell viability.*** ***Panels A & B*** show the heat map illustrating the cytotoxic effect of sertaconazole and oxiconazole (2.5–20 μM) in combination with antibiotics (2.5–20 μM) on the A549 cells, respectively. The cells were treated with sertaconazole or oxiconazole (2.5–40 μM) in combination with antibiotics (2.5–40 μM) for 24 h while growing at 37°C and 5% CO₂. The data reported correspond to the mean viability of three independent readings ± SD.

**
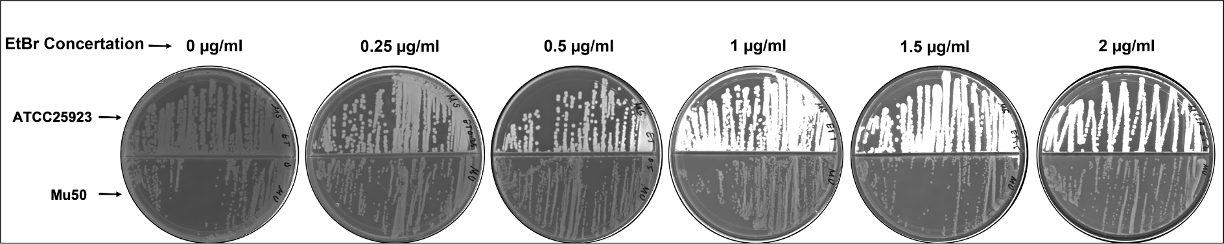
**

***Figure S2: EtBr agar cartwheel of ATCC25923*** ***and Mu50.*** ***Figure S2*** shows the fluorescence of different concentrations of EtBr in ATCC25923 and Mu50 cells after 18 hours of growth. Higher EtBr fluorescence corresponds to higher cytoplasmic retention of EtBr and lesser efflux.

**
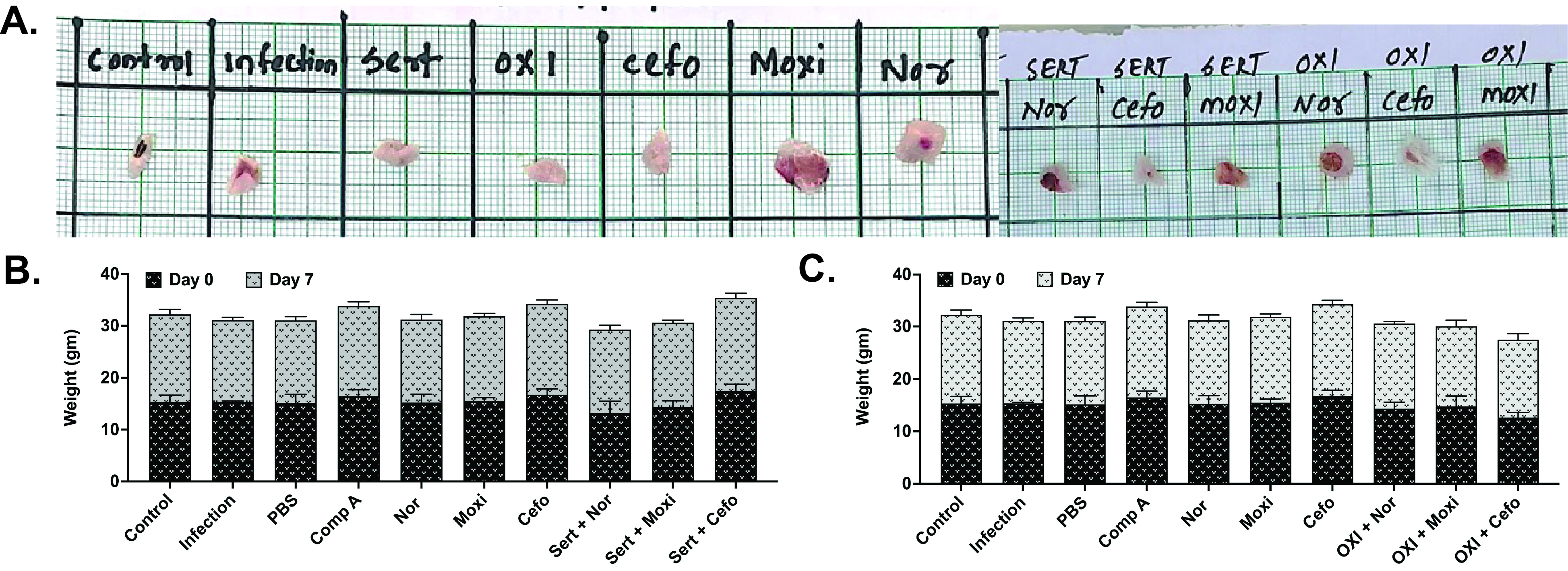
**

***Figure S3:*** ***Mice infection area and mice weight on day 1 and day 8.*** ***Panel A*** shows the collected skin tissues to determine the bacterial burden by CFU plating. ***Panel B*** shows the weights of mice on days 1 and 8, respectively. PBS: phosphate buffered saline, pH 7.4; Control: no infection; Infection: Mu50-infected cells; Sert: sertaconazole; Oxi: oxiconazole; Nor: norfloxacin; Mox: moxifloxacin; Cefo: cefotaxime.

**
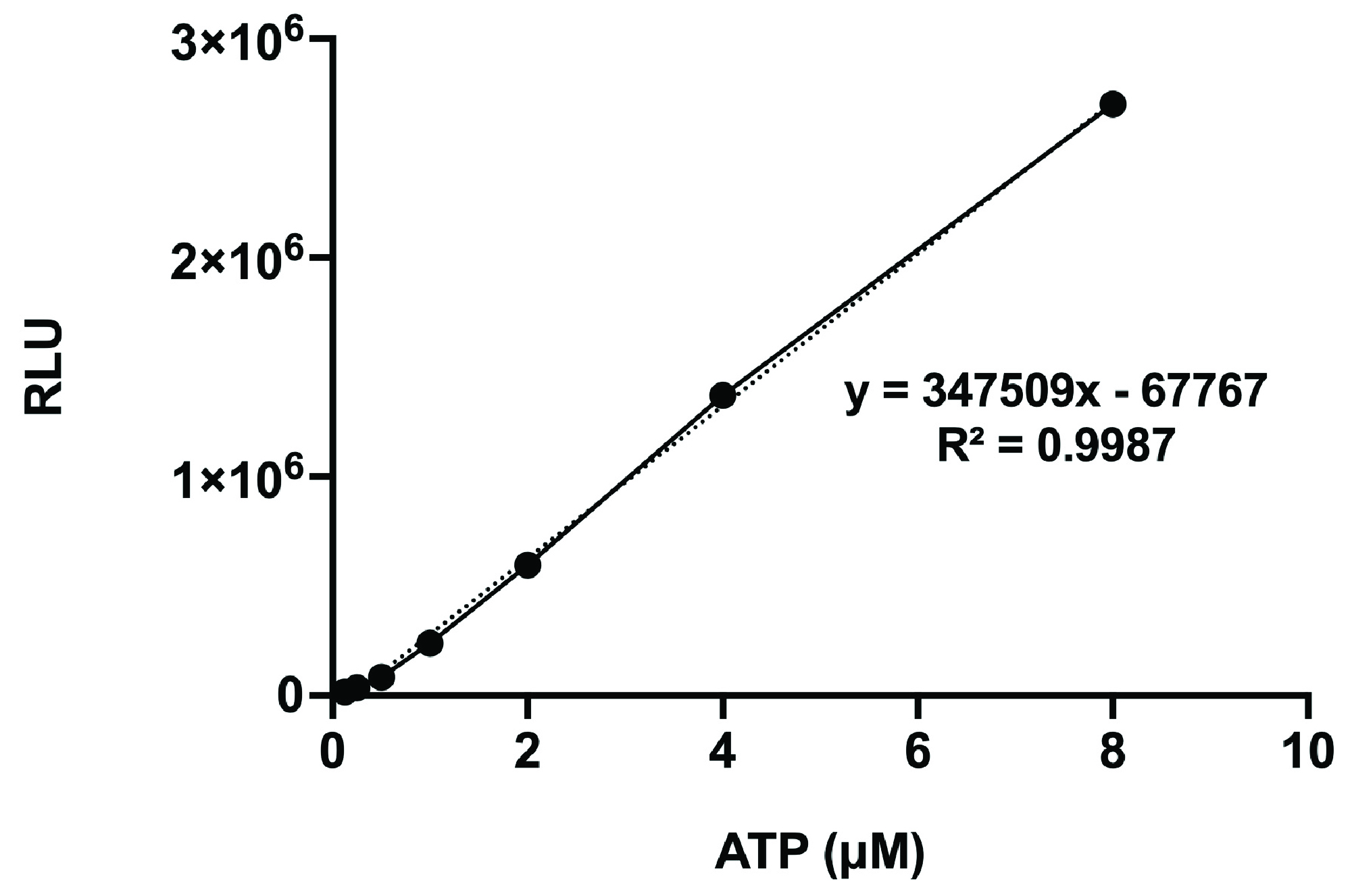
**

***Figure S4: ATP standard curve.*** The ATP standard curve was generated using the relative luminescence unit (RLU) of different ATP concentrations determined using BacTiter Glo (Invitrogen) and then plotting the RLU values for non-linear regression analysis.
